# Effects of Extracellular Resistance on Neuronal Sensitivity Under Weak Alternating Electric Field Stimulation: A Computational Study

**DOI:** 10.3390/biomimetics11040264

**Published:** 2026-04-10

**Authors:** Xiangyu Li, Shuaikang Zheng, Chunhua Yuan, Xianwen Gao

**Affiliations:** 1 School of Automation and Electrical Engineering, Shenyang Ligong University, Shenyang 110159, China; 2 College of Information Science and Engineering, Northeastern University, Shenyang 110819, China

**Keywords:** hippocampal CA3 pyramidal neurons, external electric field modulation, dual-compartment neuron model with extracellular resistance, spike sensitivity analysis

## Abstract

Weak alternating electric fields are widely used in neuromodulation techniques such as transcranial alternating current stimulation (tACS), yet the precise biophysical mechanisms underlying neuronal responses remain incompletely understood. Current computational models often neglect the electrical properties of the extracellular microenvironment, limiting their predictive accuracy. Motivated by experimentally observed frequency-dependent modulation of neuronal activity, we developed a two-compartment model of hippocampal CA3 pyramidal neurons in which extracellular resistance is explicitly parameterized and systematically examined as a key factor influencing neuronal response properties under external electric fields. Within a dual-compartment Hodgkin–Huxley framework, the neuron is divided into a “soma–basal dendrite unit” and an “apical dendrite unit,” accounting for voltage polarization induced by external fields. Using phase-locking ratio curves and three-dimensional parameter response surface, we systematically characterized neuronal sensitivity to field parameters and examined how potassium equilibrium potential ($V_{K}$) and extracellular resistance ($R_{\mathrm{out}}$) modulate these responses. Our results demonstrate that increasing $R_{\mathrm{out}}$ enhances neuronal responsiveness to external fields, while $V_{K}$ variations primarily regulate intrinsic excitability. These findings provide mechanistic insights into the frequency-dependent modulation of neuronal responses under weak electric fields, consistent with phenomena observed in biological neural systems, and provide a mechanistic and theoretical framework for understanding the joint effects of electric field amplitude and frequency on neuronal sensitivity to weak electric fields, which may help inform future neuromodulation strategies.

## 1. Introduction

Neurons are the fundamental units of natural intelligence, serving as information receivers, transmitters, and processors in the brain and peripheral nervous system [1]. When stimulated, neurons generate sequences of action potentials, where different spike patterns encode different external stimuli, a process known as neural information coding. Neural coding is a core computational function of the nervous system, allowing neurons to represent and process information through spike trains [2]. As the foundation of neural computation, this coding mechanism must be both efficient and robust, and the plasticity of firing patterns is essential for maintaining this capacity [3,4,5]. Recent studies have shown that exogenous electromagnetic stimulation, such as transcranial direct and alternating current stimulation (tDCS/tACS), can modulate neuronal firing properties by altering extracellular potentials, thereby affecting neural information processing [6,7,8]. These findings provide a theoretical basis for the clinical use of transcranial electric and magnetic stimulation techniques, which have been successfully applied to treat neurological and psychiatric disorders and pain syndromes [9]. Since the neuromodulatory effect arises primarily from electric-field–induced changes in extracellular potentials, understanding how these fields regulate neuronal firing is of significant scientific importance.

Because electric fields influence neural activity via induced potentials in the extracellular medium, accurately characterizing the electrical properties of this medium is essential for computational modeling. Substantial progress has been made in modeling the interaction between electric fields and neurons. For example, Bikson et al. investigated how uniform DC fields modulate hippocampal slice excitability [10]; Reato et al. demonstrated that low-intensity stimulation modulates both population firing rates and spike timing [7]. Grill emphasized the importance of tissue electrical properties in nerve excitation models, and Wei et al. developed a CA3 pyramidal neuron model incorporating ephaptic interactions [11,12]. However, most existing studies rely on point neurons or simplified single-compartment models, limiting the ability to capture spatially distributed field effects. Researchers have optimized classical models: Atherton et al. performed bifurcation analysis on a two-compartment pyramidal cell model, while Ilieș et al. predicted how network heterogeneity influences oscillatory dynamics [13,14,15]. Focusing on hippocampal CA3 pyramidal neurons, this study develops a single-neuron model incorporating extracellular electrical coupling and systematically analyzes neuronal dynamics under external electric field stimulation. A frequency–amplitude sensitivity-mapping approach is employed to quantify firing responses to external fields, and parameter modulation is used to identify the key determinants of sensitivity. Based on dual coding theory—where both precise spike timing and mean firing rate carry information [3,4,5]—we adopt the mean firing rate as the primary metric to achieve two objectives: (1) to characterize the firing sensitivity of single neurons under external electric fields, and (2) to determine how model parameters regulate this sensitivity.

This work systematically investigates the electrophysiological behavior of hippocampal pyramidal neurons under electric fields. Section 2 constructs a two-compartment model as the foundation. Extracellular medium effects are then incorporated to establish a field-effect model. Section 3 focuses on neuronal sensitivity to alternating electric fields, analyzing key parameters including the potassium reversal potential $V_{K}$ and extracellular resistance $R_{\mathrm{out}}$, revealing their effects on neuronal responses and providing new theoretical insight.

## 2. Materials and Methods

**Figure 1:**
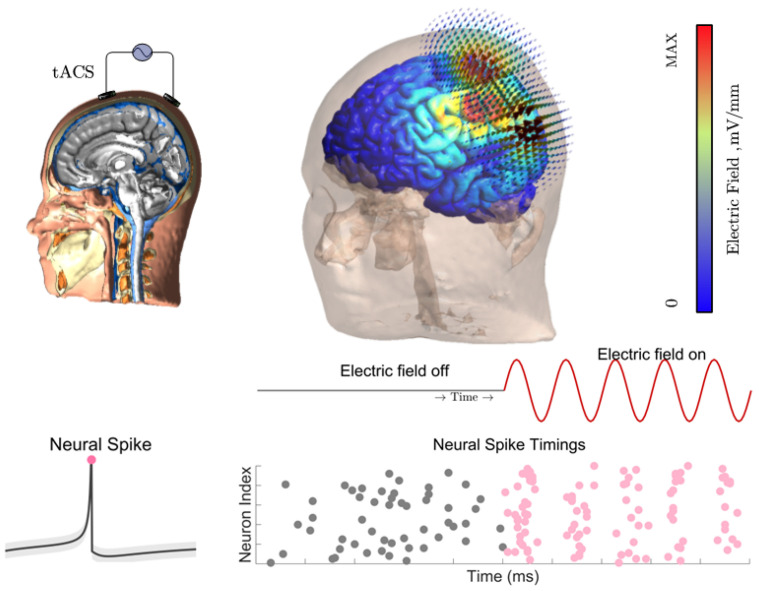
Principlesof transcranial alternating current stimulation (tACS), intracranial electric field distribution, and modulation of neuronal spiking. tACS delivers weak alternating currents through electrodes placed on the scalp, allowing currents to penetrate the scalp, skull, and other tissues to reach the brain. The induced intracranial electric field constitutes the effective dose for neuromodulation and typically attenuates in deeper regions. Left: schematic illustration of neuronal action potentials. Right: spike timing diagram showing that weak alternating electric fields bias the temporal alignment of neuronal firing while preserving the overall firing rate at stimulation intensities commonly used in human studies. Color represents the intracranial electric field strength. Gray dots indicate neurons in the resting state, whereas pink dots represent neurons in the firing state.

Transcranial alternating current stimulation (tACS) is a noninvasive brain stimulation technique whose core principle is to generate oscillatory electric fields in the brain via stimulation electrodes attached to the scalp. The applied currents propagate through the scalp, skull, and cerebrospinal fluid before ultimately reaching the brain tissue [16]. Owing to the low electrical conductivity of the skull, a large proportion of the current is shunted through the scalp, resulting in a substantial attenuation of the intracranial electric field. A key advantage of tACS lies in its flexibility for targeted neuromodulation through the adjustment of stimulation parameters. By optimizing electrode configurations (Figure 1), stimulation can be preferentially directed toward specific cortical regions such as the motor and sensory cortices. From a neuronal perspective, when neurons are exposed to electric fields induced by tACS, spatial polarization emerges along their morphology, with different neuronal processes undergoing depolarization and hyperpolarization. The dynamic evolution of such polarization can lead to the entrainment of endogenous brain rhythms to the external stimulation signal (Figure 1). These periodic polarization fluctuations directly modulate neuronal excitability and thereby influence the timing of action potential generation. In addition, complex nonlinear interactions exist between the applied electric field and ongoing spontaneous neuronal activity.

### 2.1. Standard Model

The unique spatial configuration of hippocampal pyramidal neurons provides an ideal model for investigating electromagnetic field effects. These neurons exhibit a characteristic pyramidal morphology composed of basal dendrites, soma, and apical dendrites, aligned longitudinally to form a highly ordered laminar structure. Such geometric organization significantly enhances their sensitivity to external electric fields [17,18]. Experimental observations indicate that extracellular electric fields with different orientations induce heterogeneous polarization across somatic and dendritic regions, suggesting that at least two spatially decoupled compartments are required when constructing electromagnetic field effect models [10,19].

In studies of hippocampal pyramidal neurons, a typical two-compartment modeling strategy decomposes the cell structure into a “soma–basal dendrite complex’’ and an “apical dendrite trunk’’, connected by a series resistance to represent electrical coupling. This simplified architecture preserves the essential spatial features of the neuron while effectively capturing the compartment-specific responses to external fields: the soma region, enriched with sodium and potassium channels, determines the threshold for action potential generation, whereas the apical dendrite operates primarily through passive electrotonic spread to support distal signal integration. Importantly, its membrane potential is directly modulated by the amplitude and orientation of external electric fields [13,20,21].

Given this structural specialization, hippocampal CA3 pyramidal neurons serve as an ideal model system for investigating the effects of weak electric fields. The pronounced compartment-specific polarization induced by external fields in these neurons allows for clear observation of the underlying mechanisms. Importantly, the core mechanism revealed by this model—namely, the compartment-specific polarization and its modulation by extracellular resistance—is expected to generalize qualitatively to other pyramidal neuron populations that share similar elongated morphologies, such as cortical pyramidal neurons. In such cells, the same biophysical principles apply, although the magnitude of field effects may differ quantitatively due to variations in morphological parameters (e.g., somatic and dendritic dimensions, branching patterns) or ion channel distributions. For neurons with more compact or symmetric geometries, such as many inhibitory interneurons, the field-induced polarization is likely to be smaller, but the qualitative dependence on extracellular resistance and compartmental asymmetry remains relevant. Thus, while the present study focuses on CA3 pyramidal neurons to establish a clear mechanistic understanding, the framework and insights are broadly applicable across neuronal types with appropriate parameter adjustments.

**Figure 2:**
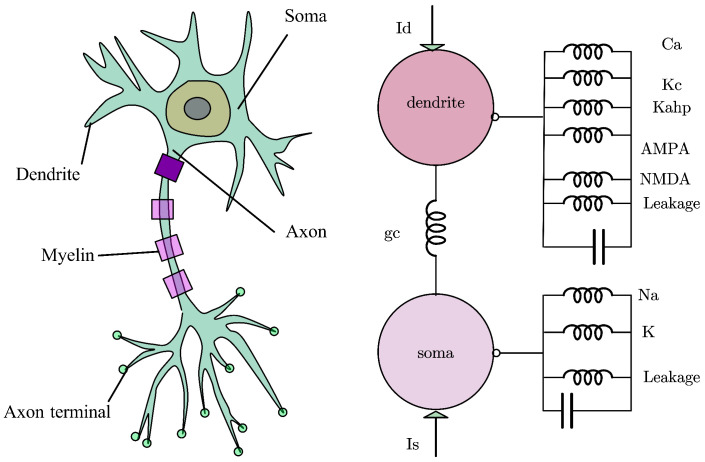
Schematicillustration of a neuron and the equivalent circuit of ionic channels in a two-compartment hippocampal pyramidal cell.

A two-compartment model was established for a single pyramidal neuron, as illustrated in Figure 2, to analyze its firing behavior under external electric fields. The neuron is divided into soma and dendritic compartments [22]. The cable equations of the PR model are given as follows:

(1) $$\begin{array}{cc} C_{m}{\dot{V}}_{s} & =-I_{s\mathrm{leak}}-I_{\mathrm{Na}}-I_{\mathrm{KDR}}+\frac{I_{DS}^{in}}{p}+\frac{I_{s}}{p},\\ C_{m}{\dot{V}}_{d} & =-I_{d\mathrm{leak}}-I_{\mathrm{Ca}}-I_{\mathrm{KAHP}}-I_{\mathrm{kc}}-I_{\mathrm{syn}}-\frac{I_{DS}^{in}}{1-p}+\frac{I_{d}}{1-p}. \end{array}$$

$V_{s}$ and $V_{d}$ denote the somatic and dendritic membrane potentials, respectively. $I_{s}$ and $I_{d}$ represent the input stimulus currents applied to the soma and dendrite. Based on the Hodgkin–Huxley (H–H) framework, the ionic currents of each channel are controlled by gating variables, and the ionic current equations are given as follows:

$$\begin{array}{cc} I_{DS}^{in} & =g_{C}\cdot \left(V_{d}-V_{s}\right),\\ I_{s\mathrm{Leak}} & =g_{L}\cdot \left(V_{s}-V_{L}\right),\\ I_{d\mathrm{Leak}} & =g_{L}\cdot \left(V_{d}-V_{L}\right),\\ I_{Na} & =g_{Na}\cdot m_{\infty }^{2}\cdot h\cdot \left(V_{s}-V_{Na}\right),\\ I_{KDR} & =g_{KDR}\cdot n\cdot \left(V_{s}-V_{K}\right),\\ I_{KC} & =g_{KC}\cdot c\cdot \chi \left(Ca\right)\cdot \left(V_{d}-V_{K}\right),\\ I_{Ca} & =g_{Ca}\cdot s^{2}\cdot \left(V_{d}-V_{Ca}\right),\\ I_{KAHP} & =g_{KAHP}\cdot q\cdot \left(V_{d}-V_{K}\right),\\ I_{NMDA} & =\frac{g_{NMDA}\;S_{i}\left(t\right)}{1+0.28\;\exp\;\left(-0.062(V_{d}-60)\right)}\;\left(V_{d}-V_{syn}\right),\\ I_{AMPA} & =g_{AMPA}\cdot W_{i}\left(t\right)\cdot \left(V_{d}-V_{syn}\right),\\ I_{syn} & =I_{NMDA}+I_{AMPA}. \end{array}$$

The ion channel kinetics on the soma and dendrite are identical, but the conductance densities differ, resulting in fast and slow currents. The soma contains three fast currents: the sodium current $I_{Na}$, the delayed rectifier potassium current $I_{KDR}$, and the leak current $I_{s,\mathrm{Leak}}$. The dendrite contains six slow currents: the calcium current $I_{Ca}$, the long-duration calcium-dependent potassium current $I_{KAHP}$, the short-duration calcium- and voltage-dependent potassium current $I_{KC}$, the leak current $I_{d,\mathrm{Leak}}$, the slow synaptic channel current $I_{NMDA}$, and the fast synaptic channel current $I_{AMPA}$. $I_{DS}^{in}=g_{c}\cdot \left(V_{d}^{in}-V_{S}^{in}\right)$ represents the current between the two compartments, where $g_{c}$ is the coupling conductance between compartments, and $\left(V_{d}^{in}-V_{S}^{in}\right)$ is the voltage difference between the two internal compartments. *p* denotes the proportion of the soma in the total membrane area. The dynamics of the gating variables for each ion channel are expressed as follows:

(2) $$\frac{dy}{dt}=\frac{y_{\infty }\left(U\right)-y}{\tau_{y}\left(U\right)}$$

where

$$y_{\infty }=\frac{\alpha_{y}}{\alpha_{y}+\beta_{y}},\;\tau_{y}=\frac{1}{\alpha_{y}+\beta_{y}},\;y=h,n,s,c,q,$$

and α and β are the corresponding rate functions (see Table 1 for details).

The dynamical evolution of intracellular Ca^2+^ concentration is given by:

(3) $$\dot{Ca}=-0.13I_{Ca}-0.075Ca$$

(4) $$\chi \left(Ca\right)=\min\left(\frac{Ca}{250},1\right)$$

The weights of the last two synaptic conductances (NMDA, AMPA) are given by:

(5) $${\dot{S}}_{i}=\sum_{j}H\left(V_{s}\left(j\right)-10.0\right)-\frac{S_{i}}{150}$$

(6) $${\dot{W}}_{i}=\sum_{j}H\left(V_{s}\left(j\right)-20.0\right)-\frac{W_{i}}{2}$$

where the summation is over all neuronal synapses, and H(x) is a step function:

(7) $$H\left(x\right)=\left\{\begin{array}{cc} 1, & x\geq 0\\ 0, & x<0 \end{array}\right.$$

Other PR model parameters are shown in Table 2.

### 2.2. Neuron Model Under External Electric Fields

Given that environmental electromagnetic fields induce extracellular potentials through the conductive properties of the surrounding medium, the equivalent circuit model in this study explicitly incorporates the composite resistive–capacitive characteristics of the extracellular medium [23,24]. For extremely low-frequency electromagnetic stimulation, the capacitive component of the medium can be neglected under direct-current and quasi-static field conditions, resulting in a simplified dual-compartment equivalent circuit. To accurately characterize transmembrane ionic kinetics, the Hodgkin–Huxley channel mechanism is adopted to describe the electrophysiological properties of each compartment, while synaptic inputs are simplified as membrane-current boundary conditions according to computational neuroscience principles [25]. Overall, this formulation establishes a hippocampal pyramidal neuron model capable of effectively capturing spatial polarization effects induced by external electric fields.

**Figure 3:**
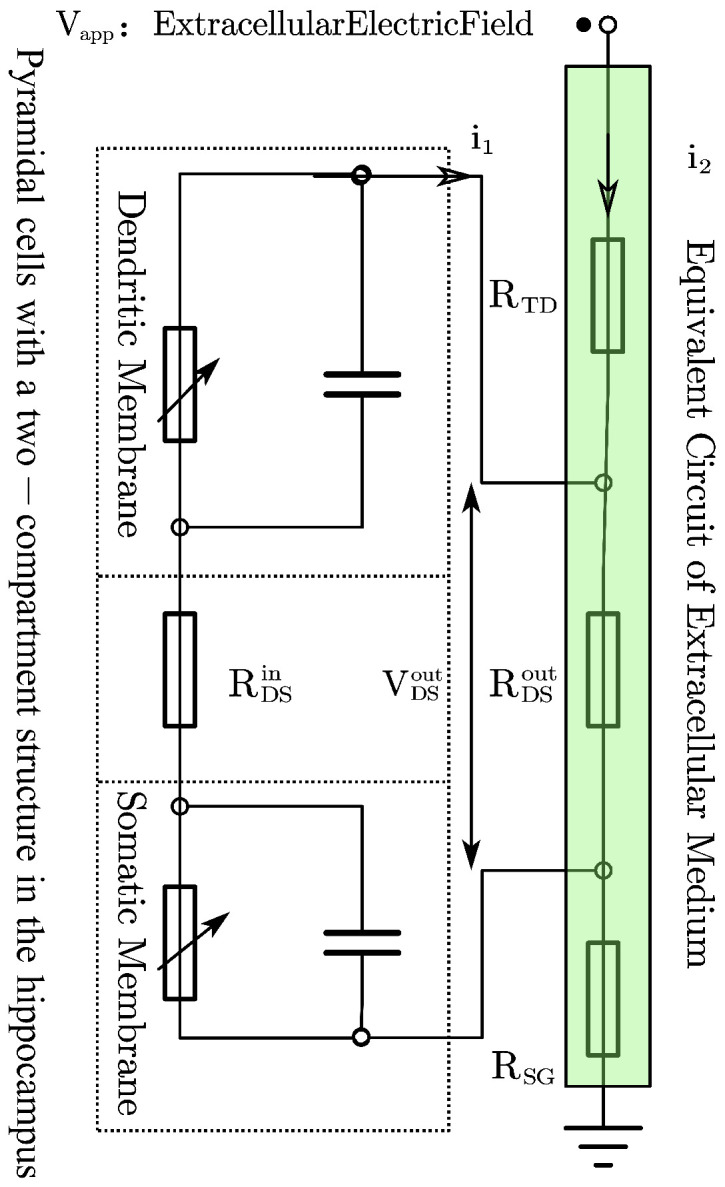
Equivalentcircuit model of a single hippocampal pyramidal neuron under an external electric field.

In the equivalent circuit representation of the proposed neuron model (Figure 3). the externally applied electric field induces a potential difference $V_{DS}^{out}$ across the neuron via the extracellular resistive pathway. Meanwhile, variations in the neuronal membrane potential feed back to modify the voltage across this extracellular resistance. Therefore, the term $V_{DS}^{out}$ is incorporated into the driving term of the intracellular coupling current:

(8) $$I_{DS}^{in}=g_{c}\cdot \left(V_{d}+V_{DS}^{out}-V_{s}\right)$$

Based on the above descriptions, the final neuronal model used in this study is described by the following equations. By incorporating the extracellular resistance mechanism defined in Section 2.2 Equation (8) into the two-compartment model in Section 2.1 Equation (1), the modified membrane potential dynamics are obtained as follows:

(9) $$\begin{array}{cc} C_{m}{\dot{V}}_{s} & =-I_{\mathrm{sleak}}-I_{\mathrm{Na}}-I_{\mathrm{KDR}}+\frac{g_{c}\cdot \left(V_{d}+V_{\mathrm{DS}}^{\mathrm{out}}-V_{s}\right)}{p}+\frac{I_{s}}{p},\; \end{array}$$

(10) $$\begin{array}{cc} C_{m}{\dot{V}}_{d} & =-I_{\mathrm{dleak}}-I_{\mathrm{Ca}}-I_{\mathrm{KAHP}}-I_{\mathrm{Kc}}-I_{\mathrm{syn}}-\frac{g_{c}\cdot \left(V_{d}+V_{\mathrm{DS}}^{\mathrm{out}}-V_{s}\right)}{1-p}+\frac{I_{d}}{1-p}. \end{array}$$

The definitions of all ionic current terms (e.g., $I_{\mathrm{Na}}$, $I_{\mathrm{KDR}}$, $I_{\mathrm{Ca}}$) are identical to those given in Section 2.1 and are not repeated here.

Here, $V_{DS}^{out}$ denotes the voltage across the extracellular resistance $R_{DS}^{out}$. According to Kirchhoff’s current law, the circuit currents satisfy:

(11) $$\left\{\begin{array}{c} i_{1}\left(R_{DS}^{in}+R_{DS}^{out}\right)+i_{2}R_{DS}^{out}=V_{s}-V_{d},\\ i_{1}R_{DS}^{out}+i_{2}\left(R_{TD}+R_{SG}+R_{DS}^{out}\right)=V_{e} \end{array}\right.$$

Solving Equation (11) yields the extracellular voltage contribution:

(12) $$V_{DS}^{out}=\left(i_{1}+i_{2}\right)R_{DS}^{out}$$

External electric fields are commonly represented as voltage sources applied to the extracellular domain of the neuron. Under sinusoidal weak-field stimulation, the electric field varies with time as:

(13) $$E\left(t\right)=E_{0}\sin\left(\omega t\right)$$

where $E_{0}$ is the amplitude of the electric field intensity (mV/mm), ω=2πf is the angular frequency, and *f* denotes the field frequency. Under the parallel-plate electrode approximation, the applied field can be considered spatially uniform, forming a linear potential gradient along the neuronal axis. Let *d* denote the effective geometric distance between the soma and the dendrite; then, the extracellular potential difference induced by the field is:

(14) $$\Delta V_{\mathrm{ext}}\left(t\right)=E\left(t\right)\;d=E_{0}d\sin\left(\omega t\right)$$

This potential difference, measured in millivolts, directly reflects the electric-field–induced variation in the extracellular potential. The membrane potential of the neuron is defined as the difference between intracellular and extracellular potentials:

(15) $$V_{\mathrm{mem}}\left(t\right)=V_{\mathrm{intra}}\left(t\right)-V_{\mathrm{ext}}\left(t\right)$$

Thus, the field-induced $\Delta V_{\mathrm{ext}}\left(t\right)$ directly alters the transmembrane voltage difference, thereby modulating membrane polarization. In the circuit representation, the external electric field can be equivalently modeled as an AC voltage source applied between the corresponding extracellular nodes:

(16) $$V_{E}\left(t\right)=A\sin\left(\omega t\right)$$

where the amplitude *A* is defined as:

(17) $$A=E_{0}d$$

This expression is formally consistent with the voltage excitation in the circuit, facilitating implementation in numerical models. The equivalent voltage source drives transmembrane current through the extracellular resistance network.

For the selection of extracellular resistance values in the model, this paper is based on hippocampal slice experiments between plates and refers to relevant theoretical models [10,19,26]. In the experiment, the distance *d* between the two plates is approximately 5 mm, and the length of a single hippocampal pyramidal neuron is approximately 200 μm. Assuming the extracellular space is a uniform medium, we thus obtain the ratio between extracellular resistances as shown in Table 3. The extracellular resistance in the model is represented by $R_{\mathrm{DS}}^{\mathrm{out}}$, which reflects the effective resistive pathway in the extracellular space surrounding the neuron. Physiologically, the properties of the extracellular medium are influenced by changes in extracellular potassium concentration ${\left[K^{+}\right]}_{o}$. Experimental studies have shown that increasing ${\left[K^{+}\right]}_{o}$ induces cellular swelling, which reduces the extracellular volume fraction and decreases the effective cross-sectional area of the extracellular space. Because electrical resistance is inversely proportional to the cross-sectional area of the conducting medium (R∝1/A), this reduction in extracellular volume leads to an increase in the effective extracellular resistance.

To incorporate this physiological effect, the extracellular resistance is defined relative to the intracellular dendro–somatic resistance $R_{\mathrm{DS}}^{\mathrm{in}}$ through the ratio

(18) $$r=\frac{R_{DS}^{out}}{R_{DS}^{in}}=0.1+\left({\left[K^{+}\right]}_{o}-3.5\;\mathrm{mM}\right)\times 0.01$$

Here $R_{\mathrm{DS}}^{\mathrm{in}}$ denotes the intracellular coupling resistance between dendritic and somatic compartments and is inversely proportional to the coupling conductance $g_{c}$. Using the given inter-compartmental conductance $g_{c}=2.1\;{S/\mathrm{cm}}^{2}$ and total membrane area $\mathrm{Area}=6\times 10^{-6}\;{\mathrm{cm}}^{2}$, we substitute into:

(19) $$R_{\mathrm{DS}}^{\mathrm{in}}=\frac{1}{g_{c}\cdot \mathrm{Area}}=\frac{1}{2.1\times 6\times 10^{-6}}\approx 80\;M\Omega .$$

Based on experimentally observed variations in extracellular ionic conditions, the ratio *r* is assumed to vary between 0.1 and 0.15 as ${\left[K^{+}\right]}_{o}$ increases from approximately 3.5 mM to 8.5 mM. For this fixed value of $R_{\mathrm{DS}}^{\mathrm{in}}$, this range implies that the extracellular resistance $R_{\mathrm{DS}}^{\mathrm{out}}$ varies from approximately 8 MΩ to 12 MΩ, corresponding to an increase of about 33%.

Such variations in extracellular resistance are consistent with experimental measurements showing that increases in ${\left[K^{+}\right]}_{o}$ reduce extracellular volume in hippocampal CA1 and CA3 pyramidal neuron regions, thereby increasing the effective extracellular impedance [27]. Therefore, the selected range of $R_{\mathrm{DS}}^{\mathrm{out}}$ in the present model reflects physiologically plausible changes in extracellular resistive properties associated with ionic and geometric modifications of the extracellular microenvironment. Other resistances are shown in Table 3.

### 2.3. Numerical Simulation and Spike Analysis

The model equations were numerically integrated using the MATLAB R2018b stiff solver ode15s with tolerances $\mathrm{RelTol}=1\times 10^{-6}$ and $\mathrm{AbsTol}=1\times 10^{-8}$, and a maximum step size of 0.5 ms. Simulations were performed over 0–5000 ms with a sampling interval of 0.01 ms. To eliminate transient dynamics, only activity after 1000 ms was used for analysis. Action potentials were detected from the membrane potential V(t) using an adaptive peak-detection procedure based on the MATLAB R2018b findpeaks function. Detection thresholds were defined according to the voltage range of the signal, with constraints on minimum peak prominence and a minimum inter-peak interval of 3 ms to avoid multiple detections of a single spike.

The mean firing rate was calculated from the number of detected spikes within the steady-state window. Although weak electric fields primarily modulate neuronal activity by shifting spike timing, such timing shifts accumulate over long simulations and lead to systematic changes in interspike intervals. The mean firing rate therefore serves as a compact statistical descriptor of spike-train dynamics and allows robust comparison of neuronal responses across the explored parameter space.

## 3. Results

This section investigates the mechanisms underlying neuronal sensitivity to alternating current (AC) electric fields, with a particular focus on the role of the extracellular environment in mediating field–neuron interactions. In contrast to conventional models that simplify or neglect extracellular space, we explicitly incorporate two key extracellular determinants: the potassium reversal potential $V_{K}$ and the dendrite–soma extracellular resistance $R_{DS}^{out}$. The parameter $V_{K}$, determined by the extracellular potassium concentration ${\left[K^{+}\right]}_{o}$, reflects neuronal ionic homeostasis and metabolic state, whereas $R_{DS}^{out}$ characterizes the geometry and conductive properties of the extracellular space and serves as a critical physical mediator of electric field effects. Parameterizing these variables enables a systematic analysis of neuronal responses to AC electric field stimulation [28,29].

Building on this framework, we examine neuronal firing dynamics and phase-locking behavior under independent and combined modulation of $V_{K}$ and $R_{DS}^{out}$. Both parameters are physiologically and pathologically relevant: elevations in extracellular potassium concentration alter $V_{K}$ and strongly modulate neuronal excitability, contributing to epileptiform activity, while increases in extracellular resistance, such as those arising from extracellular space shrinkage, markedly enhance neuronal sensitivity to electric fields via pseudosynaptic mechanisms [30,31]. Together, these results demonstrate that neuronal sensitivity to AC electric fields is jointly constrained by intracellular ionic regulation and extracellular physical properties, providing a unified computational framework for interpreting tissue-level responses to electric field stimulation across physiological and pathological conditions.

### 3.1. Influence of $R_{\mathrm{out}}$ on AC Electric Field Sensitivity

#### 3.1.1. Influence of $R_{\mathrm{out}}$ on the Regulation of Neuronal Response by External Electric Fields

**Figure 4:**
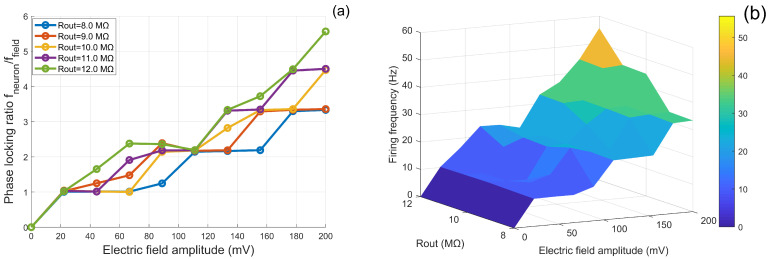
Field-dependent modulation of neuronal firing under different extracellular resistance conditions. (**a**) Phase-locking ratio as a function of electric field amplitude *A* for different values of $R_{\mathrm{out}}$. (**b**) Three-dimensional frequency–amplitude response map showing firing frequency across electric field amplitude and stimulation frequency.

Under a fixed electric-field frequency of 10 Hz, this study systematically investigates how the extracellular resistance $R_{\mathrm{out}}$, as a key intrinsic parameter, modulates neuronal responses to external field amplitudes. Using a multi-parameter scanning approach, we examine how variations in $R_{\mathrm{out}}$ within the range of 8 to 12 MΩ influence phase-locking behavior and firing rates across electric-field amplitudes of 0 to 200 mV, as illustrated in Figure 4. These results elucidate the role of $R_{\mathrm{out}}$ in shaping neuronal sensitivity to external electric fields.

At low field amplitudes (A<50 mV), the response curves diverge markedly. Neurons with larger $R_{\mathrm{out}}$ (e.g., 12.0 MΩ) exhibit a steeper rise in phase-locking ratio with increasing *A*, indicating a higher “synchronization sensitivity” to weak electric fields. In other words, only minimal external field strength is required to induce strong phase locking. In the moderate-amplitude range (50 mV <A< 150 mV), the curves display fluctuations and intersections, suggesting a complex interaction between intrinsic membrane oscillations and external field drive, where the modulatory role of $R_{\mathrm{out}}$ persists but becomes nonlinear. At high amplitudes (A>150 mV), all groups reach saturation, yet neurons with larger $R_{\mathrm{out}}$ achieve significantly higher final locking ratios (e.g., ∼6 for 12.0 MΩ vs. ∼4 for 8.0 MΩ), reflecting more efficient conversion of field input into synchronized spiking under strong stimulation.

To visualize the overall trend, Figure 4 presents the three-dimensional distribution of firing rate in the amplitude–$R_{\mathrm{out}}$ plane. Firing frequency increases prominently with both electric-field amplitude and $R_{\mathrm{out}}$, indicating that higher extracellular resistance enhances field-induced membrane depolarization and reduces excitation threshold.

As an intrinsic structural parameter, $R_{\mathrm{out}}$ modulates current partitioning between soma and dendrite, thereby regulating the driving efficiency of external electric fields on neuronal excitability. These results demonstrate that $R_{\mathrm{out}}$ affects not only resting electrical properties but also the magnitude and sensitivity of field-induced responses. This finding suggests that, at tissue or network scales, morphological and impedance heterogeneity across neurons may lead to diverse neuromodulation outcomes under external electric-field stimulation, offering a single-cell mechanistic basis for heterogeneous responses observed in brain stimulation.

#### 3.1.2. Effects of $R_{\mathrm{out}}$ on the Frequency-Dependent Modulation of Neuronal Firing by Electric Fields

**Figure 5:**
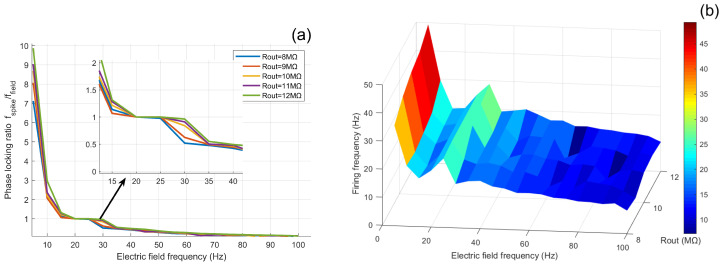
Extracellular resistance–dependent modulation of neuronal phase locking and firing dynamics. (**a**) Phase -locking ratio, defined as the ratio between neuronal firing frequency and applied electric field frequency, plotted as a function of field frequency under different values of $R_{\mathrm{out}}$. (**b**) Corresponding three-dimensional parameter response surface illustrating how firing frequency is jointly regulated by $R_{\mathrm{out}}$ and electric field frequency.

This study systematically investigates how the extracellular resistance $R_{\mathrm{out}}$ influences neuronal firing responses under alternating electric fields. With the field amplitude fixed at 100 mV, varying $R_{\mathrm{out}}$ (8–12 MΩ) and the field frequency (5–100 Hz) reveals a clear frequency-dependent effect Figure 5.

According to the two-compartment circuit analysis, $R_{\mathrm{out}}$—as a key component of the extracellular pathway—determines how effectively the external field is transmitted from the extracellular space into the dendritic and somatic compartments. At low frequencies (5–15 Hz), the membrane capacitance behaves nearly as an open circuit, and the system is dominated by resistive behavior. Increasing $R_{\mathrm{out}}$ enhances the voltage drop across the extracellular pathway, leading to a larger effective transmembrane drive and an approximately linear increase in firing rate. For example, under a 5 Hz field, the firing rate increases from 35.62 Hz at $R_{\mathrm{out}}=8$ MΩ to 49.32 Hz at $R_{\mathrm{out}}=12$ MΩ, an increase of 38.4%.

At intermediate frequencies (20–50 Hz), the membrane capacitance introduces substantial reactance, and the circuit becomes resistive–capacitive. Here, the interaction between the external field and intrinsic ion-channel dynamics becomes nonlinear, giving rise to frequency competition and phase locking. Changes in $R_{\mathrm{out}}$ alter the impedance of the extracellular pathway, thereby modifying both the amplitude and phase of the injected field signal. This shifts the balance between the external drive and intrinsic rhythms, producing multiple crossings and fluctuations in the locking-ratio curves. For instance, at 30 Hz the locking ratio increases sharply from 0.520 to 0.964, reflecting a complex nonlinear transition.

At high frequencies (>60 Hz), the capacitive reactance of the membrane becomes very small, and the membrane effectively shunts most of the external field. Consequently, the majority of the voltage drop occurs across $R_{\mathrm{out}}$, resulting in only a weak transmembrane driving component. Under these conditions, the locking ratios converge to ∼0.1 across all tested $R_{\mathrm{out}}$ values, indicating a greatly diminished dependence on $R_{\mathrm{out}}$.

The three-dimensional parameter response surface in Figure 5b further visualizes the complex firing-rate structure arising from the joint variation of $R_{\mathrm{out}}$ and field frequency, consistent with the multi-mechanism interactions governed by changes in extracellular pathway impedance.

In summary, $R_{\mathrm{out}}$ shapes neuronal responses to alternating electric fields in a frequency-dependent manner. The effects arise from linear voltage-division behavior at low frequencies, nonlinear impedance interactions and phase-locking dynamics at intermediate frequencies, and capacitive shunting at high frequencies. These results highlight the essential role of extracellular pathway impedance in determining the frequency-dependent sensitivity of neurons to weak electric fields.

### 3.2. Effect of $V_{K}$ on AC Electric Field Sensitivity

#### 3.2.1. Effect of $V_{K}$ on Amplitude-Dependent Modulation of Neuronal Firing

Under a fixed electric field frequency of 10 Hz, this study systematically investigates how the potassium reversal potential ($V_{K}$), as a key intrinsic electrophysiological parameter, modulates neuronal responses to external field amplitude. Through multi-parameter scanning, we examined the firing behavior and phase-locking characteristics of the neuron as $V_{K}$ varied from −80 mV to −40 mV and the electric field amplitude ranged from 0 to 200 mV. The results reveal the central regulatory role of $V_{K}$ in determining neuronal sensitivity to external electric fields, demonstrating that changes in the potassium electrochemical driving force directly reshape the amplitude–response profile and phase-locking performance of the neuron.

**Figure 6:**
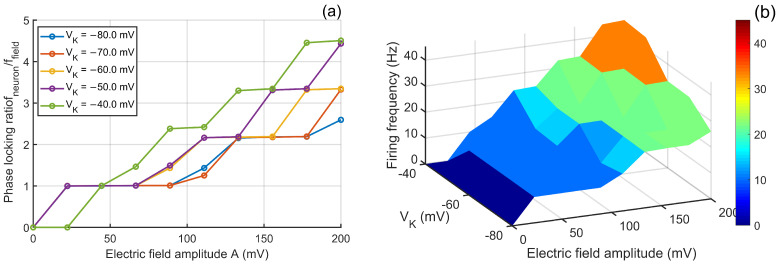
Potassium reversal potential–dependent modulation of neuronal phase locking and firing dynamics. (**a**) Phase-lockingratio as a function of electric field amplitude under different potassium reversal potentials $V_{K}$ (f=10Hz, $R_{\mathrm{out}}=10.0\;M\Omega$). (**b**) Corresponding three-dimensional parameter response surface illustrating firing frequency jointly regulated by $V_{K}$ and electric field amplitude.

Experimental results show that under different $V_{K}$ values, the threshold of phase-locking ratio jump (i.e., the electric field amplitude at which the phase-locking mode switches) exhibits significant differences. As shown in Figure 6a, when $V_{K}=-40\;\mathrm{mV}$, the phase-locking ratio jumps above 2 at an amplitude of 80 mV; in contrast, when $V_{K}=-80.0\;\mathrm{mV}$, the jump is delayed until the amplitude reaches 120 mV. This indicates that the smaller (more negative) the $V_{K}$, the more sluggish the neuron’s phase-locking response to changes in electric field amplitude, requiring higher amplitudes to trigger the phase-locking mode transition. Consequently, an increase in $V_{K}$ enhances the neuron’s sensitivity to electric field amplitude.

Regarding firing frequency characteristics, the 3D parameter response surface in Figure 6b clearly illustrates the co-regulatory effect of $V_{K}$ and electric field amplitude. Under fixed frequency conditions, firing frequency increases nonlinearly with electric field amplitude, and changes in $V_{K}$ significantly alter the dynamics of this growth curve. Notably, for the same electric field amplitude, a depolarizing shift of $V_{K}$ leads to a marked increase in firing frequency. For example, at an amplitude of 100 mV, increasing $V_{K}$ from −80 mV to −40 mV raises the firing frequency from 25 Hz to approximately 45 Hz, fully confirming the crucial role of $V_{K}$ in regulating intrinsic neuronal excitability.

From the perspective of ionic dynamics, these phenomena can be attributed to the modulation of potassium driving force by $V_{K}$. Depolarization of $V_{K}$ reduces the electrochemical driving force of potassium ions, weakening K^+^ efflux during action potential repolarization and allowing the membrane potential to more readily maintain a relatively depolarized state. This enhancement of intrinsic excitability makes the neuron more sensitive to external electric fields, manifested as a lower phase-locking threshold, higher firing frequency, and steeper response slope. Additionally, $V_{K}$ may influence membrane impedance by altering the steady-state activation properties of potassium conductance, thereby modulating the voltage drop induced by the applied field across the neuronal membrane. This provides a novel perspective for understanding the mechanisms through which $V_{K}$ regulates field sensitivity.

In summary, under fixed frequency conditions, $V_{K}$ serves as a key intrinsic regulatory parameter that significantly affects neuronal response to electric field amplitude by modulating potassium current driving force and neuronal excitability. A depolarizing shift of $V_{K}$ not only lowers the threshold amplitude required for synchronized firing but also enhances neuronal sensitivity to changes in the applied electric field. These findings provide important theoretical insights for understanding individual variability and plasticity in electric field neuromodulation.

#### 3.2.2. Effect of $V_{K}$ on the Modulation of Neuronal Firing by Electric Field Frequency

The potassium reversal potential ($V_{K}$), as a key electrochemical parameter regulating potassium channel function, plays a critical role in neuronal responses to external electric fields. In this study, by systematically scanning the $V_{K}$ parameter (from −90 mV to −20 mV) and electric field frequency (5–60 Hz) under a fixed electric field amplitude of 100 mV, we thoroughly analyzed the regulatory mechanism of $V_{K}$ on neuron–field synchronization and intrinsic excitability.

**Figure 7:**
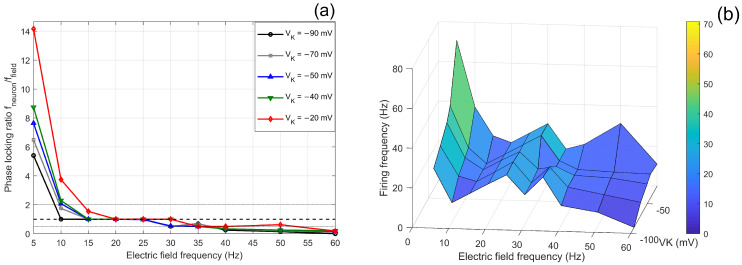
Potassium reversal potential–dependent modulation of neuronal phase locking and firing dynamics. (**a**) Phase-locking ratio of neuronal firing frequency as a function of applied electric field amplitude under different potassium reversal potentials $V_{K}$. (**b**) Corresponding three-dimensional parameter response surface illustrating firing frequency jointly regulated by $V_{K}$ and the electric field amplitude.

Experimental results indicate that $V_{K}$ significantly affects neuronal phase-locking behavior and intrinsic excitability. Figure 7a illustrates the phase-locking ratio of neuronal firing as a function of electric field frequency under different $V_{K}$ conditions. Clear differences in the 1:1 phase-locking regime (phase-locking ratio ≈1) are observed across $V_{K}$ values. Specifically, when $V_{K}=-90$ mV, stable 1:1 phase-locking occurs over a relatively broad frequency range of 10–25 Hz. As $V_{K}$ depolarizes to −70 mV and −50 mV, the phase-locking window shifts toward higher frequencies, with stable synchronization occurring in the 15–25 Hz range. When $V_{K}$ further depolarizes to −40 mV, the synchronization range expands slightly to 15–30 Hz. Under a strongly depolarized condition ($V_{K}=-20$ mV), the 1:1 phase-locking regime is primarily observed in the 20–30 Hz frequency range. Overall, these results indicate a systematic shift of the phase-locking window toward higher frequencies as $V_{K}$ becomes more depolarized. In other words, more negative $V_{K}$ values favor synchronization with lower-frequency electric fields, whereas depolarized $V_{K}$ conditions preferentially support phase-locking at mid-to-high stimulation frequencies.

Figure 7b shows the three-dimensional distribution of firing frequency under the joint action of $V_{K}$ and electric field frequency. Regarding intrinsic excitability, $V_{K}$ is positively correlated with neuronal firing frequency. Quantitative analysis reveals that the mean firing frequency is 15.3 Hz at $V_{K}=-90$ mV; 17.9 Hz at $V_{K}=-70$ mV; 18.8 Hz at $V_{K}=-50$ mV; 21.2 Hz at $V_{K}=-40$ mV; and markedly increases to 28.6 Hz at $V_{K}=-20$ mV. The 3D firing frequency parameter response surface further confirms that as $V_{K}$ depolarizes from −100 mV, the firing frequency surface rises overall, with the enhancement effect particularly pronounced in the 10–30 Hz electric field frequency range.

From the perspective of ionic kinetics, $V_{K}$ regulates neuronal excitability by altering the transmembrane driving force of potassium ions: the depolarization of $V_{K}$ reduces the difference between the potassium equilibrium potential and the membrane potential, thereby weakening the potassium outward current at the late stage of action potential repolarization. This attenuation of repolarization capacity, on the one hand, places neurons in a state of relatively longer relative refractory period or subthreshold depolarization after a single action potential, leading to an overall increase in excitability, which is manifested as an elevation in the average firing frequency (Figure 7b). On the other hand, the altered repolarization dynamics affect the response characteristics of the membrane potential to subsequent stimuli. Under hyperpolarized $V_{K}$ (e.g., -90 mV), the strong repolarizing current enables the membrane potential to reset rapidly, allowing precise tracking of low-frequency rhythms. In contrast, under depolarized $V_{K}$ (e.g., −20 mV), the weakened repolarization makes neurons more sensitive to the cumulative effect of continuous high-frequency stimulation, which facilitates reaching the threshold more easily, thus shifting their phase-locking range toward higher frequencies. However, excessively weak repolarization may also disrupt the stable tracking of intermediate frequencies, resulting in the widest phase-locking window at certain intermediate $V_{K}$ values (e.g., −40 mV), while the window narrows again at extremely depolarized $V_{K}$. This indicates that the regulation of frequency selectivity by $V_{K}$ is a result of the trade-off between repolarization strength and excitability.

In summary, the results demonstrate that the potassium reversal potential ($V_{K}$) plays a key role in modulating neuronal responses to external electric fields. By altering the transmembrane driving force of potassium ions, $V_{K}$ regulates repolarization dynamics and intrinsic neuronal excitability. More negative $V_{K}$ values promote stronger repolarizing $K^{+}$ currents, maintaining neurons in a relatively low-excitability state that supports stable synchronization with lower-frequency electric fields. In contrast, depolarized $V_{K}$ reduces the potassium driving force and increases neuronal excitability, shifting the frequency range of stable phase-locking toward higher stimulation frequencies.

These findings highlight that neuronal sensitivity to external electric fields depends not only on the properties of the applied stimulation but also on intrinsic membrane parameters that shape neuronal firing dynamics. In this framework, $V_{K}$ influences the effective synchronization window by regulating intrinsic excitability and firing frequency. This mechanism may contribute to variability in neuronal responses across different physiological states and provides theoretical insight for optimizing parameter selection in brain electric field stimulation protocols.

## 4. Discussion

Weak extracellular electric fields have been shown experimentally to influence neuronal activity at both the subthreshold and spiking levels [32]. Electrophysiological studies using cortical slice preparations have demonstrated that endogenous or externally applied electric fields with amplitudes on the order of 1–2 mV/mm can induce measurable membrane polarization while modulating ongoing network oscillations [29]). In these experiments, uniform electric fields were applied across cortical slices using parallel electrodes, allowing controlled investigation of field–neuron interactions under physiologically relevant conditions. Such findings provide direct evidence that weak extracellular fields can alter neuronal membrane potentials without directly triggering action potentials.

Beyond subthreshold effects, weak electric fields can also modulate spike timing when neurons receive synaptic input [19] demonstrated that small electric fields can significantly shift spike timing in hippocampal pyramidal neurons, reflecting the nonlinear amplification of field-induced membrane polarization near firing threshold. In addition, oscillatory electric fields have been reported to influence firing phase and neuronal synchronization, suggesting that weak fields can entrain neuronal activity and modulate spike timing relative to the field phase [7]).

The results of the present computational study are consistent with these experimental observations. Our model reproduces key features reported in electrophysiological studies, including field-induced membrane polarization, modulation of firing activity, and phase-dependent changes in neuronal responses. Furthermore, by explicitly incorporating extracellular resistance as a model parameter, our results suggest that variations in the extracellular microenvironment may significantly influence neuronal sensitivity to weak electric fields, providing a potential mechanistic explanation for variability observed in experimental studies. This study reveals the influence of electric fields on neuronal firing sensitivity and analyzes the roles of dendrite–soma extracellular resistance ($R_{\mathrm{out}}$) and potassium reversal potential ($V_{K}$) in regulating neuronal sensitivity to external electric fields.

### 4.1. Circuit-Level Mechanisms Associated with the Extracellular Resistance ($R_{\mathrm{out}}$)

This study reveals the critical role of the extracellular resistance $R_{\mathrm{out}}$ in determining neuronal sensitivity to electric field stimulation. Unlike traditional views that attribute the field effect solely to membrane polarization, our results indicate that $R_{\mathrm{out}}$ regulates the proportion of extracellular current shunted between the soma and dendrites, thereby modulating the coupling strength between membrane polarization and ion channel dynamics. This mechanism is fundamental in shaping neuronal field sensitivity and frequency response characteristics. Our findings are consistent with the tissue resistivity theory proposed by Grill [10], but refine this concept from the tissue level to the single-neuron scale.

When $R_{\mathrm{out}}$ increases, extracellular current pathways are restricted, forcing more field energy through transmembrane routes, which significantly enhances the depolarization effect in the somatic compartment. This mechanism explains experimental observations: with higher $R_{\mathrm{out}}$, the membrane potential perturbations induced by the applied field are more effectively retained in the soma, amplifying ion channel activity during early depolarization and allowing neurons to achieve phase-locking at lower field amplitudes. This trend is particularly evident in the low-amplitude field range, manifesting as enhanced phase-locking sensitivity, and in high-field conditions, it results in higher ultimate phase-locking ratios and peak firing frequencies. Notably, in the medium-field range, the phase-locking curves of different $R_{\mathrm{out}}$ groups show fluctuations and intersections, suggesting a competition between intrinsic membrane oscillatory dynamics and external driving forces. This phenomenon indicates that electric field regulation is not a simple linear input-output process but is shaped by multiscale dynamical factors, including membrane time constants, dendrite–soma conduction delays, and phase relationships of channel kinetics.

Based on these findings and current gaps in the field, future work should focus on several directions: (1) integrating neuronal morphological diversity into multiscale computational models to systematically study how different dendritic architectures influence $R_{\mathrm{out}}$-mediated modulation; (2) developing novel probes capable of real-time monitoring of extracellular microenvironment changes to validate the dynamic variation of $R_{\mathrm{out}}$ under physiological and pathological conditions; (3) combining clinical imaging data to explore correlations between individual brain tissue conductivity differences and neuromodulatory efficacy, providing a theoretical foundation for precision medicine; and (4) investigating how the nervous system optimizes electric signal processing through structural feature modulation during development and plasticity, opening new avenues for understanding mechanisms underlying learning and memory.

### 4.2. Modulatory Role of $V_{K}$ in Shaping Ion-Channel Responses to External Electric Fields

This study systematically analyzed neuronal responses to external electric fields under different $V_{K}$ conditions, revealing the central role of the potassium reversal potential in neural modulation. The results not only validate the working hypothesis that $V_{K}$ shapes neuronal excitability by modulating the potassium driving force, but also highlight its additional roles in frequency selectivity and phase-locking stability.

At the molecular level, our results confirm that $V_{K}$ directly influences the repolarization process of action potentials by altering the potassium current reversal potential, consistent with classical Hodgkin–Huxley model predictions [10]. The main contribution of this study is to reveal how $V_{K}$ modulates the dynamical response properties of single neurons under external electric field stimulation. Specifically, depolarizing shifts of $V_{K}$ not only increase neuronal excitability but also systematically alter phase-locking behavior and frequency selectivity. As $V_{K}$ shifts from −90 mV to −20 mV, the 1:1 phase-locking range migrates from 10–25 Hz to 20–30 Hz, indicating that $V_{K}$ may optimize neuronal responses to rhythmic external inputs by modulating membrane time constants. Compared with previous studies, while Reato et al. [7] proposed that electric fields could modulate network dynamics by adjusting population firing rates, our study further reveals that this modulation is profoundly influenced by $V_{K}$. Notably, the synergistic interaction between $V_{K}$ and electric field frequency provides a mechanistic explanation for heterogeneous responses of different brain regions to identical electrical stimuli—distinct neuronal populations may express different potassium channel subtypes, leading to variations in effective $V_{K}$ and thus frequency selectivity.

From a broader physiological and pathological perspective, our findings offer a new framework for understanding diverse neural phenomena. First, neuronal plasticity observed during learning and memory may partly result from changes in potassium channel expression and function, which alter effective $V_{K}$ and optimize neural network information processing. Second, under pathological conditions, such as extracellular potassium accumulation during epileptic seizures, changes in $V_{K}$ may significantly enhance neuronal synchrony, providing novel insights into the initiation and propagation of epileptic activity.

### 4.3. Translational Implications and Future Directions

The present model provides mechanistic insight into how extracellular resistance modulates neuronal responses to weak electric fields, which has potential implications for neuromodulation techniques such as transcranial alternating current stimulation (tACS). In non-invasive stimulation, intracranial electric fields are typically on the order of ∼0.2–1 mV/mm and induce subthreshold membrane polarization in cortical and hippocampal neurons [33]. Multiple studies have shown that low-frequency stimulation (<20 Hz) produces more pronounced effects than high-frequency stimulation (50–100 Hz). Although these fields are weak, experimental and computational studies have demonstrated that they can influence spike timing, oscillatory dynamics, and neuronal excitability [34,35]. Notably, it has been found that alternating electric fields as low as 0.3 mV/mm can modulate the activity of mammalian neurons in awake and behaving states [36]. Our results suggest that the electrical properties of the extracellular environment, particularly extracellular resistance, can substantially alter the effective coupling between applied fields and membrane polarization. This finding indicates that tissue microstructure may play an important role in determining neuronal sensitivity to stimulation, which could be relevant when optimizing stimulation parameters such as field amplitude, frequency, and orientation while remaining within established safety limits.

In the present study, the amplitude range used in the simulations extends beyond typical physiological electric field strengths. This choice was made to systematically explore the dynamical response landscape of the neuron model and to identify transition regimes in the parameter space. In vivo, tACS-induced electric fields are typically on the order of 0.2–1 mV/mm, corresponding to sub-millivolt membrane polarization. Therefore, the lower-amplitude region of the parameter space explored in this study is more directly relevant to physiological conditions. Combined with the aforementioned evidence of minimum effective dose, further exploration of the dynamical responses in this region is of great significance for determining the minimum effective dose of tACS and optimizing stimulation protocols.

An important feature of the present framework is that the key parameters governing neuronal sensitivity to external electric fields are defined through measurable structural and physiological variables rather than neuron-specific constants. In particular, the extracellular resistance $R_{\mathrm{out}}$ represents the effective resistive pathway in the extracellular space surrounding the neuron. This quantity depends on neuronal morphology, including dendritic extent and soma–dendrite spatial arrangement, as well as on the conductivity and volume fraction of the extracellular medium. Consequently, neurons with different dendritic architectures or embedded in distinct extracellular environments are expected to exhibit different effective $R_{\mathrm{out}}$ values. Similarly, the potassium reversal potential $V_{K}$ is determined by ionic concentrations through the Nernst equation. Because intracellular potassium concentration varies across neuron types and physiological conditions, $V_{K}$ can be independently parameterized using experimentally measured values reported in the literature. Together, these formulations allow the model to be adapted to other neuron classes by substituting the corresponding morphological and ionic parameters while preserving the same dynamical structure. While the present study focuses on a single-neuron biomimetic model, the framework can be extended to larger neuronal populations. Previous studies have shown that weak electric fields can modulate network-level activity, including synchronization and oscillatory entrainment, even when individual neurons experience only small membrane perturbations [37,38]. Embedding the proposed neuron model into network simulation frameworks would therefore enable investigation of how extracellular resistance influences collective dynamics under field stimulation, potentially revealing mechanisms underlying field-induced modulation of brain rhythms.

Experimental validation of the model predictions could be pursued using in vitro hippocampal slice preparations, where controlled electric fields can be applied while monitoring neuronal responses with intracellular or patch-clamp recordings. Such experiments would allow direct testing of predicted relationships between extracellular electrical properties and membrane polarization or spike timing modulation.

Finally, future work may integrate the present neuron-scale model with realistic head models derived from anatomical imaging. Computational tools that estimate electric field distributions in the human brain based on individual anatomy are increasingly used to guide neuromodulation protocols [39]. Coupling these macroscopic electric field simulations with biophysically grounded neuron models may provide a pathway toward patient-specific neuromodulation optimization, enabling more precise prediction of neuronal responses to externally applied stimulation.

## 5. Conclusions

This study shows that the modulation of neuronal firing sensitivity by external electric fields is a complex process involving multiple scales and mechanisms. The field parameters (amplitude and frequency) nonlinearly regulate firing rates and phase-locking patterns by altering the degree of membrane depolarization and interacting with intrinsic neuronal oscillations, with specific parameter ranges (50–150 mV amplitude, 30–60 Hz frequency) producing the highest sensitivity. The dendrite–soma extracellular resistance ($R_{\mathrm{out}}$) shapes neuronal sensitivity and frequency response by controlling how extracellular currents are partitioned between soma and dendrites, which determines the distribution of field-induced energy across the membrane. The potassium equilibrium potential ($V_{K}$), as a key ionic-level regulator, modifies the repolarization process of action potentials by shifting the potassium reversal potential and influences neuronal frequency selectivity and population synchronizability.These findings provide important theoretical insights into the cellular mechanisms underlying electric field–based neuromodulation and characterize the sensitivity of neuronal responses to key model parameters under weak electric fields.

## Figures and Tables

**Table 1:** Ratefunctions for gating variables in the CA3 pyramidal neuron model.

Gating Variable	Forward Rate Function	Backward Rate Function
Activation of $Na^{+}$ channel; *m*	$\alpha_{m}=\frac{0.32(-13.1-V_{s})}{\exp(\left(-13.1-V_{s}\right)/4)-1}$	$\beta_{m}=\frac{0.28(V_{s}-40.1)}{\exp(\left(V_{s}-40.1\right)/5)-1}$
Inactivation of $Na^{+}$ channel; *h*	$\alpha_{h}=0.128\exp\left(\frac{17.0-V_{s}}{18.0}\right)$	$\beta_{h}=\frac{4}{1+\exp(\left(-40.0-V_{s}\right)/5)}$
Activation of $K^{+}$ channel; *n*	$\alpha_{n}=\frac{0.016(35.1-V_{s})}{\exp(\left(35.1-V_{s}\right)/5)-1}$	$\beta_{n}=0.25\exp\left(0.5-0.025V_{s}\right)$
Activation of $Ca^{2+}$ channel; *s*	$\alpha_{s}=\frac{1.6}{1+\exp(-0.072\left(V_{d}-65\right))}$	$\beta_{s}=\frac{0.02(V_{d}-51.1)}{\exp(\left(V_{d}-51.1\right)/5)-1}$
Activation of $Ca^{2+}$-sensitive $K^{+}$ channel; *c*	$\alpha_{c}=\left\{\begin{array}{c} \frac{\exp(\left(V_{d}-10.0\right)/11-\left(V_{d}-6.5\right)/27)}{18.975},\\ 2\exp\left(\frac{65-V_{d}}{27}\right) \end{array}\right.$	$\beta_{c}=\left\{\begin{array}{cc} 2\exp\left(\frac{6.5-V_{d}}{27}\right)-\alpha_{c}, & V_{d}\leq 50.0\\ 0, & V_{d}>50.0 \end{array}\right.$
Activation of $K^{+}$ AHP channel; *q*	$\alpha_{q}=\min\left(0.00002Ca,0.01\right)$	$\beta_{q}=0.001$

Notes: Rate functions are defined for gating variables *m*, *h*, *n*, *s*, *c*, *q*.

**Table 2:** Parameter values of the PR model.

Parameter	Value
*p*: Proportion of soma membrane area	0.5
Total membrane area	$6\times 10^{-6}\;{\mathrm{cm}}^{2}$ (soma radius 5 μm)
$g_{c}$: Coupling conductance between compartments	$2.1\;{\mathrm{mS}/\mathrm{cm}}^{2}$
$C_{m}$: Membrane capacitance	$3.0\;\mu {F/\mathrm{cm}}^{2}$
**Reversal potentials (mV), reference: −60 mV**	
$V_{Na}$	120
$V_{Ca}$	140
$V_{K}$	−38.56
$V_{L}$	0
$V_{syn}$	60
**Channel conductances (${\mathrm{mS}/\mathrm{cm}}^{2}$)**	
$g_{L}$	0.1
$g_{Na}$	30
$g_{KDR}$	15
$g_{Ca}$	10
$g_{KAHP}$	0.8
$g_{KC}$	15
$g_{NMDA}$	0.03
$g_{AMPA}$	0.0045
**Stimulus currents ($\mu {A/\mathrm{cm}}^{2}$)**	
$I_{s}$	0
$I_{d}$	0.7

Conductances and reversal potentials follow the original PR model definition.

**Table 3:** Ratios of resistances in the equivalent extracellular network.

Name	Formula/Value	Description
Dendrite–Soma	$R_{DS}^{out}=r\;R_{DS}^{in}$	Extracellular resistance between dendrite and soma
Field–Dendrite	$R_{TD}=12\;R_{DS}^{out}$	Resistance between positive field terminal and dendrite
Soma–Ground	$R_{SG}=12\;R_{DS}^{out}$	Resistance between soma and ground

*r* denotes the scaling factor relating intracellular and extracellular resistances.

## Data Availability

The original contributions presented in the study are included in the article, further inquiries can be directed to the corresponding author.

## Abbreviations

The following abbreviations are used in this manuscript:

tACS	Transcranial alternating current stimulation
tDCS	Transcranial direct current stimulation
CA3	Cornu Ammonis area 3 (hippocampal subregion)
$V_{K}$	potassium equilibrium potential
$R_{\mathrm{out}}$	Extracellular resistance
DC	Direct current
AC	Alternating current
